# Evolutionary design of two-dimensional material Fabry–Perot structures for enhanced second harmonic generation

**DOI:** 10.1515/nanoph-2022-0459

**Published:** 2022-12-15

**Authors:** Rabindra Biswas, Asish Prosad, Lal A. S. Krishna, Sruti Menon, Varun Raghunathan

**Affiliations:** Department of Electrical Communication Engineering, Indian Institute of Science, Bangalore 560012, India

**Keywords:** Fabry Perot cavities, gallium selenide, genetic optimization, second harmonic generation

## Abstract

The integration of two-dimensional (2D) materials with resonant photonic structures is seen as a promising direction for enhancing its nonlinear optical response. The design of such heterogeneous resonant structures has often relied on multi-parameter sweeps to determine the optimized dimensions of resonant optical structure that results in good resonance characteristics, often in the absence of the 2D material. Such an approach is computationally intensive and may not necessarily result in efficient generation or collection of nonlinear signals from the designed structure. Here, we report hybrid-genetic optimization (HGA) based design and experimental demonstration of second harmonic generation (SHG) enhancement from Fabry–Perot structures of single and double multilayer gallium selenide (GaSe) flakes with bottom silicon dioxide, and index matched polymethyl methacrylate spacer/encapsulation layers. HGA technique utilized here speeds up the multilayer cavity design by 8.8 and 89-times for the single and double GaSe structures when compared to the full parameter-sweep, with measured SHG enhancement of 128- and 400-times, respectively, when compared to a reference sample composed of GaSe layer of optimized thickness on 300 nm silicon dioxide layer. SHG conversion efficiencies obtained from the HGA structures are 1–2 orders of magnitude higher than previous reports on 2D material integrated resonant metasurfaces or Bragg cavities.

## 1 Introduction

Two-dimensional (2D) layered materials are known to exhibit strong nonlinear optical effects with unique layer dependence [1, 2] and opto-electronic tunability [3], making them promising candidates for realizing next generation active photonic devices. The nonlinear optical processes studied in 2D material systems include wavelength conversion, saturable absorption, optical modulation, parametric down-conversion etc. [1]. Second harmonic generation (SHG) is one such well-known wavelength conversion processes which involves the parametric up-conversion of high-intensity incident fundamental laser to twice its frequency. The SHG process is routinely used as a tool for identifying layer number, crystallographic orientation [2], twist angle [4], for visualizing grain boundaries and defects [5] in 2D material nanosheets. While the inherent nonlinear optical susceptibility values for the 2D materials are notably high [1], the overall SHG conversion efficiency still tends to be low for any useful photonic application. Excitonic resonances are useful for enhancing nonlinear optical processes [2, 6], [7], [8], [9]. However, excitonic resonances being inherently narrowband limit the useful spectral window for observing strong SHG response. The reduced interaction length can be mitigated using multilayer 2D materials. SHG response is however sensitive to crystal symmetry, resulting in strong layer number dependence [2]. The complete cancellation of SHG response for anti-parallel orientation of transition metal dichalcogenides (TMDC) results in negligible SHG response from even numbered layers [2, 10]. This can be circumvented using non-standard crystal polytypes such as 3R-polytype for TMDCs [11], *ε*-form for gallium/indium selenide [12–15], and lattice-distorted rhenium disulfide layers [16].

Another promising route towards SHG enhancement for photonic device applications is the integration of 2D materials with resonant photonic structures. Resonant structures in the form of photonic crystals [17], ring resonators [18], distributed Bragg reflector cavities [19–22], plasmonic structures [23, 24] and resonant optical metasurfaces [25, 26] have been integrated with 2D materials. Multi-fold SHG enhancement from 2D materials have been demonstrated when excited near high quality factor quasi bound states in continuum resonances [27–30]. However, such hybrid structures still exhibit low conversion efficiencies due to limited interaction lengths, higher order diffraction effects and strong optical absorption from the underlying resonant structure at the nonlinear signal wavelength. The design of such heterogeneous resonant structures has often relied on “brute force” multi-parameter sweeps to determine suitable device dimensions for the resonant structure with good linear optical characteristics, in the absence of the 2D material. Such approaches are both computationally intensive and may not necessarily result in designs that achieve the highest possible conversion efficiencies. It would be beneficial to develop co-design approaches in which the resonant structures are designed in the presence of the 2D material with the design process driven with the objective of maximizing the detected nonlinear signal. This strategy naturally lends itself to finding structures with the highest SHG conversion efficiency number, which is more meaningful when compared to resonant enhancement factors.

In this paper, we present one such co-design approach for designing 2D material based muti-layer, Fabry–Perot (FP) structures using a hybrid genetic optimization algorithm (HGA) with the objective of maximizing the overall SHG response at the detector plane. Evolutionary optimization algorithms have been used in the past for enhancing Mie scattering from silicon nanostructure [31] and for increasing four wave mixing response from metallic mesh structures [32]. Topological optimization has also been used in the design study of doubly resonant multicavity structure based on alternating AlGaSe/Al_2_O_3_ thin-film layers for SHG enhancement [33]. To the best of our knowledge, the present work is the first demonstration of the use of genetic optimization and experimental demonstration applied to the design of 2D-material based photonic structures. Here we focus on the design of multilayer FP-type structures owing to the ease of fabrication, simpler light coupling, broad wavelength range of operation and the SHG polarization properties determined solely by the 2D materials rather than the resonant structure.

2D material samples for optoelectronic studies are often prepared on silicon dioxide (SiO_2_) film with thickness in the range of 250–300 nm to obtain good visual contrast [34]. The optimum SiO_2_ thickness for best visual contrast does not necessarily result in the highest nonlinear optical signal generation. There is a clear need to determine the individual layer thicknesses in the stack with the end application taken into consideration. In this direction, there are previous studies on enhancing nonlinear response from monolayer TMDCs, graphene layers with suitable SiO_2_ thickness [35] and metal back-plane with dielectric spacers [23]. Such studies determine the optimum layer thickness by relying on simplified analytical models or multi-parameter sweeps which are difficult to scale to more complex multilayer stacks.

Here we focus on HGA based optimization of two different FP structures with single or double gallium selenide (GaSe) on SiO_2_ film with polymethyl methacrylate (PMMA) used as both spacer and encapsulation layers. PMMA is a good index matching layer to the bottom SiO_2_ and offers the added benefit of encapsulating the 2D material, with the potential for preventing deterioration of the 2D material due to continuous ambient exposure [36]. The utility of the evolutionary optimization algorithm in accelerating the optimization of 2D material multilayer stacks when compared to multi-parameter sweep is best illustrated for the case of the double GaSe FP structure for which four different layer thicknesses are to be optimized and a full parameter-sweep becomes particularly time consuming. Most notably, the HGA technique reduces the computation time by ∼8.8 and 89-times for the single and double FP GaSe structure design, respectively, when compared to the full parameter space sweep with sequential execution of the electromagnetic simulations. Experimental studies on the optimized single and double GaSe FP structures shows 128 and 400-times SHG enhancement, respectively, when compared to the reference sample showing good agreement with the design studies, with 1–2 orders of magnitude increase in SHG conversion efficiency when compared to hybrid resonant photonic structures [22, 25, 26]. The reference sample used here for comparison is a GaSe layer of the same thickness as that of the HGA optimized design on 300 nm thick SiO_2_ layer on silicon (Si) substrate, due to the widespread use of such stacks for 2D material optical studies.

## 2 Design studies

**Figure 1:**
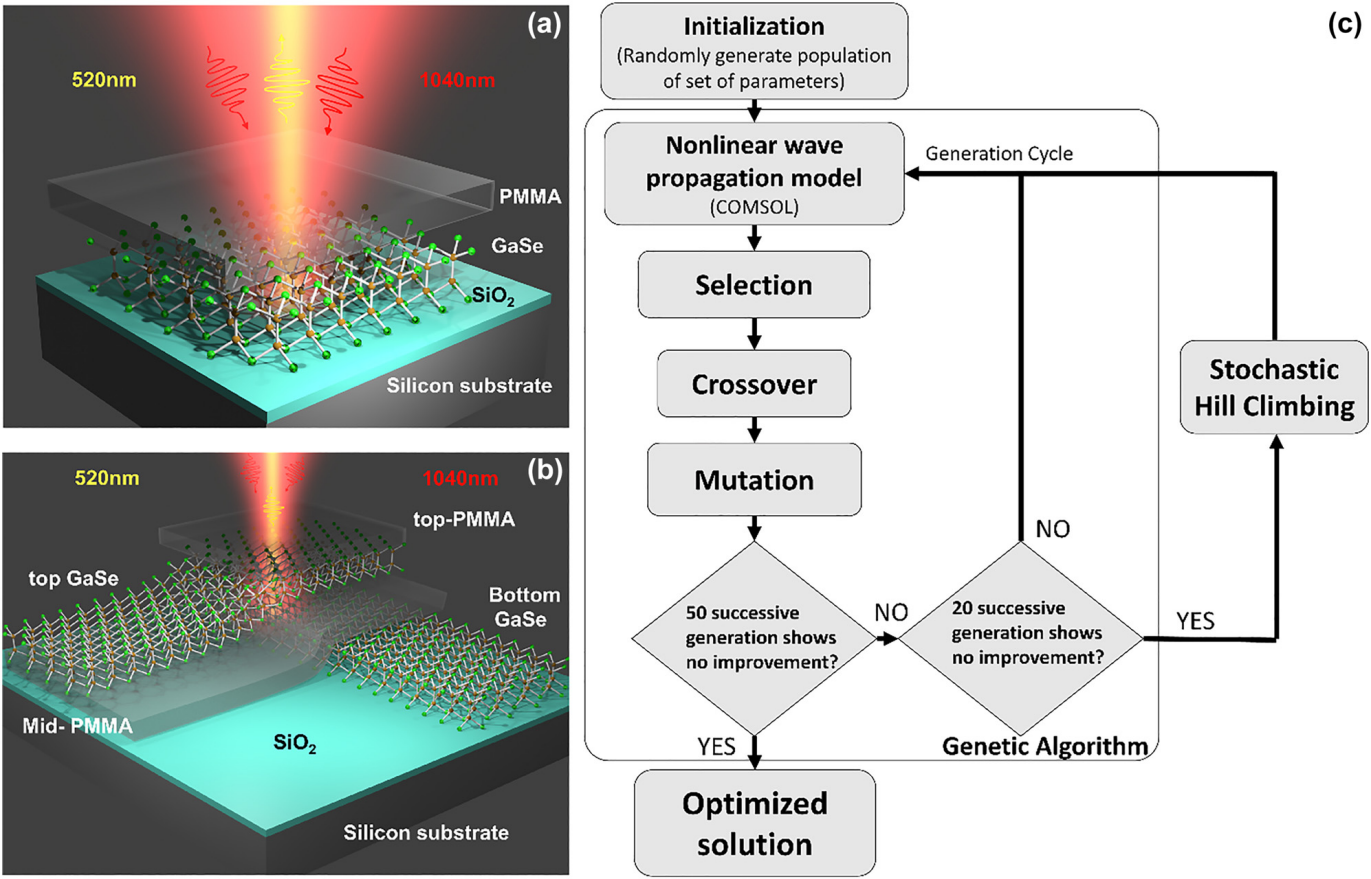
Schematic of: (a) single GaSe FP structure consisting of PMMA encapsulated multilayer GaSe on SiO_2_/Si substrate and (b) double GaSe FP structure consisting of two multilayer GaSe on top of SiO_2_/Si substrate with low index PMMA spacer and encapsulation layer. (c) Flowchart of HGA utilized here for the design combining GA and SHC routines.

Figure 1 shows the schematic of the 2D material-based FP structures considered in this study with the multilayer GaSe sandwiched between bottom SiO_2_ film on Si substrate and PMMA spacer/encapsulation layers. The crystallographic axes of the top and bottom GaSe layers for the double GaSe FP structure are assumed to be aligned such that the nonlinear dipole oscillators from the two spatially separated layers are parallel to each other. Such alignment can be performed using SHG polarization studies performed separately on the two GaSe layers, as discussed below. GaSe is an indirect band-gap semiconductor [12] that belongs to the family of metal monochalcogenides. *ε*-polytype of GaSe exhibits D_3h_ symmetry with the following non-vanishing second order nonlinear optical susceptibility elements [2, 13]: $\chi_{yyy}^{\left(2\right)}=-\chi_{yxx}^{\left(2\right)}=-\chi_{xxy}^{\left(2\right)}$ = $-\chi_{xyx}^{\left(2\right)}=80\pm 18pm/V$ , with *x* and *y* referring to the zig-zag and arm-chair axis, respectively. *ε*-GaSe is known to exhibit layer independent non-centrosymmetry which results in SHG emission irrespective of the layer number [13]. Further, GaSe shows strong non-resonant nonlinear response over a broad wavelength range above its bandgap wavelength of ∼610 nm, making it a promising material for realizing photonic devices across a wide transparency window spanning visible to mid infrared wavelengths.

The HGA technique utilized in this work combines genetic optimization algorithm (GA) [37] and stochastic hill climbing (SHC) [38] with the objective of determining the optimum layer parameters (i.e. thicknesses) that maximizes the overall SHG at the detector plane. The SHG signal strength is computed for each HGA iteration for the different thickness parameters using nonlinear wave propagation simulations. The nonlinear wave propagation model is discussed in the methods section.

A flow-chart of the HGA utilized in this work with the condition used for transition from GA to SHC and the final convergence to the optimized parameters is shown in Figure 1(c). GA is an evolutionary global optimization technique that imitates the natural selection process that occurs in the process of evolution of biological species, i.e., through selection, cross-over and mutation. It is used here for fast parameter space search to determine solutions close to the desired objective. SHC is a local search algorithm that uses randomness as part of the search process for accelerating the convergence to the desired objective. The HGA implemented in this work is discussed in detail in the methods section.

**Figure 2:**
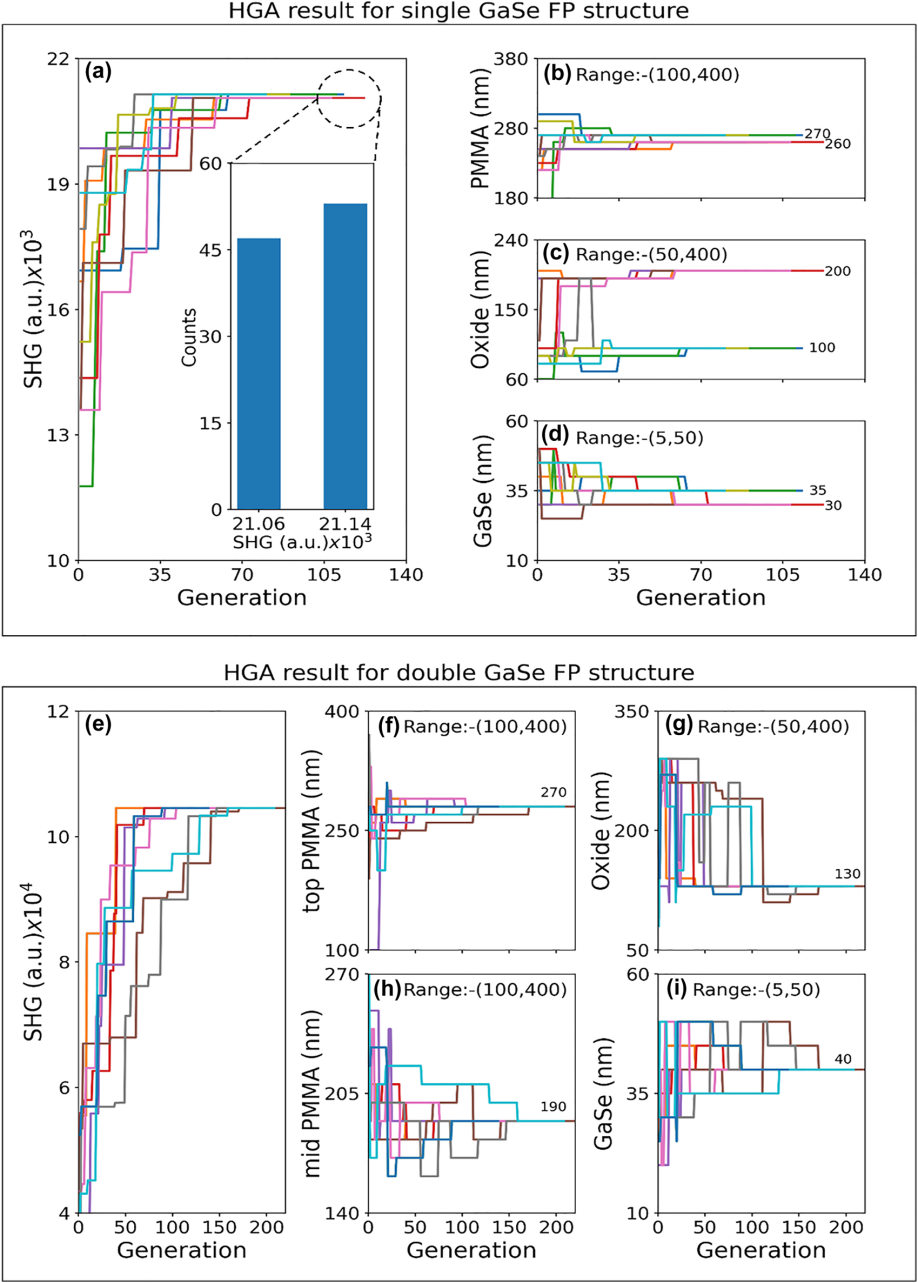
Summary of the HGA optimization results. (a) SHG signal for single GaSe FP structure, and optimized thickness values for: (b) PMMA layer, (c) SiO_2_ layer and (d) multilayer GaSe as a function of iteration number of the HGA optimization process. The results of HGA are shown for ten independent runs. Histogram showing the SHG signal for the optimized structure for 100 independent simulation runs is shown in the inset of (a). (e) SHG signal for double GaSe FP structure and thickness of: (f) top PMMA layer, (g) SiO_2_ layer, (h) mid PMMA spacer layer and (i) multilayer GaSe as a function of iteration number of the HGA optimization process. The results of HGA are shown for ten independent runs. The thickness range and optimum values obtained are specified in (b–d) and (f–i).

Figure 2 summarizes the results of HGA optimization for the single and double GaSe FP structures. The HGA routine is repeated for ten independent runs to ascertain if the HGA results converge to global SHG maxima. For the single GaSe FP structure, the HGA optimized solution is found to converge to two separate solutions with the SHG signal strength differing by about 2.5%, with the thickness of the top PMMA/GaSe/bottom SiO_2_ layers obtained as: 270 nm/35 nm/100 nm and 260 nm/30 nm/200 nm, respectively, as shown in Figure 2(a)–(f). A histogram of the SHG signal strength for 100 such independent HGA runs is also shown in the inset of Figure 2(a) with the number of occurrences of the two optimized solutions obtained as 46 and 54, with the higher SHG signal design occurring slightly more frequently. It is known that achieving absolute global maxima is not always guaranteed when using evolutionary optimization algorithms [37]. In the present study, given the small variation in the optimized SHG signal strength across two widely spaced parameters, the evolutionary algorithm is found to converge to one of the two solutions. We have also calculated the SHG signal strength for all possible thickness values using a full parameter space sweep which is shown as contour maps in Figure S2 of the Supplementary Material. For the single GaSe FP structures considered here, the full parameter space sweep is possible in reasonable time scales, albeit slower than the HGA approach. Two distinct maxima with comparable SHG signal were observed for SiO_2_ layer thickness of 100 and 200 nm. Similar double-peaked SHG as a function of varying SiO_2_ layer thickness has been reported for monolayer MoS_2_ with an order of magnitude variation in SHG with SiO_2_ thickness variation [35].

The design studies of single GaSe FP structures clearly showed that arbitrary increase in 2D material thickness does not translate to an increase in SHG signal. The optimum GaSe layer thickness that results in highest SHG is determined by the phase mismatch consideration between the interacting fundamental/SHG waves and optical absorption effects at the wavelengths of interest [10, 39]. In this context, quasi-phase matching is a well-known technique to further increase nonlinear signal by mitigating the reversal of energy flow due to phase mismatch using periodic domains of opposite orientation of nonlinear crystals [40]. Here, we explore a related technique to increase the SHG signal by utilizing two aligned GaSe layers separated by suitable low index spacer layer that can potentially enhance the overlap between the GaSe nonlinear media and the fundamental field. The target thickness parameters for the double GaSe FP structure are the bottom SiO_2_, two multilayer GaSe, PMMA spacer and encapsulation layers. The thickness of the top and bottom GaSe are kept identical to simplify the design process. Figure 2(e)–(i) summarizes the HGA results for the double GaSe FP structure for ten independent HGA runs, with the range of thicknesses considered indicated in each plot. The final optimized thickness parameters obtained for the PMMA encapsulation/top GaSe/PMMA spacer/bottom GaSe/SiO_2_ layers are: 270 nm/40 nm/190 nm/40 nm/130 nm, respectively. The ten independent HGA runs consistently converge to the same solution, pointing to the convergence to single global SHG maxima. A sensitivity analysis on the optimized designs taking into consideration the realistic variation in the thickness parameters during sample preparation, is discussed in Figures S4 and S5 of the Supplementary Material. A comparison of the computation time for the HGA and full parameter space search for the two optimized structures is also shown in Table T3 of the Supplementary Material. The computation time is estimated to be reduced by ∼8.8 and ∼89-times for the HGA when compared to the full parameter space search. This points to the clear benefit of using HGA for fast convergence to the best FP structure design especially when the multilayer stack increases in complexity.

## 3 Experimental results

**Figure 3:**
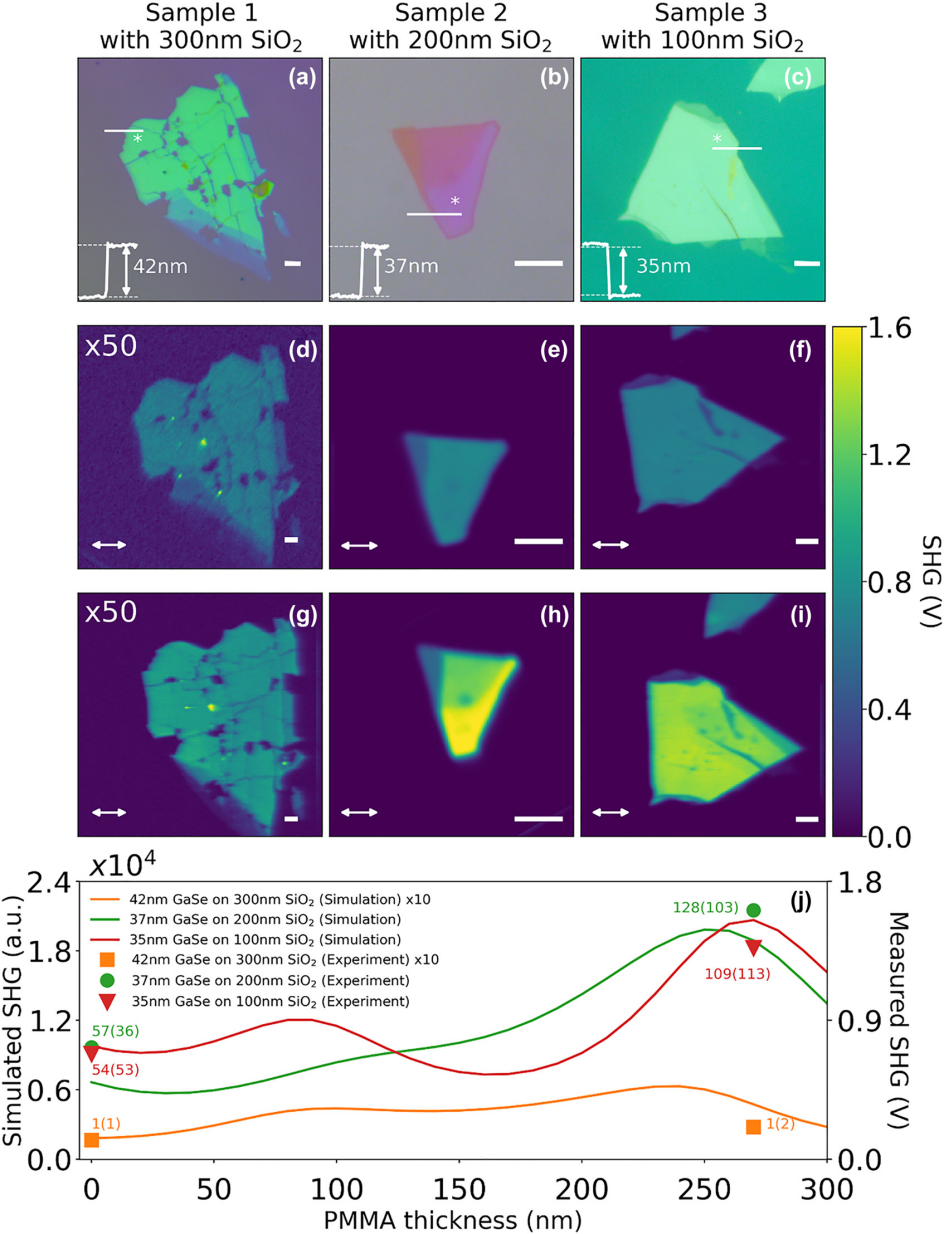
Summary of the experimental studies on the single GaSe FP structure Optical image of: (a) S1 (reference sample): 42 nm GaSe/300 nm SiO_2_, (b) S2: 37 nm GaSe/200 nm SiO_2_ and (c) S3: 35 nm GaSe/100 nm SiO_2_. Thickness of each flake in the region of interest indicated by the AFM line profile shown as an inset. (d–f) SHG images without PMMA coating for samples S1 to S3. (g–i) SHG image with PMMA coating for samples S1 to S3. (j) Comparison between experimental SHG data points for samples S1 to S3, and simulation results showing SHG signal as a function of PMMA layer thickness. Scale bar for all optical and SHG image correspond to 5 µm. The input polarization of the fundamental is indicated by the double-sided arrow in the SHG image. SHG enhancement factors for experiments (simulation) are indicated next to the experimental data points.

For the experimental demonstration of SHG enhancement from single GaSe FP structure, three samples with varying bottom SiO_2_ thickness were prepared to study the SHG dependence on underlying SiO_2_ thickness. The sample preparation for the single GaSe FP structures is discussed in the methods section. Figure 3(a)–(c) shows the optical images of the three samples labelled as S1, S2 and S3 with GaSe/SiO_2_ layer thickness of 42 nm/300 nm, 37 nm/200 nm and 35 nm/100 nm, respectively. GaSe thickness in the range of 30–40 nm are identified in each sample using atomic force microscopy (AFM) imaging, with the corresponding height profiles for the chosen locations shown as insets. SHG measurements are performed using a nonlinear optical microscope system, as described in the methods section. A femtosecond pulsed fiber laser centered at 1040 nm wavelength is used as the fundamental excitation source, with the SHG centered at 520 nm wavelength spectrally selected using band-pass filters before detection using a photon-multiplier tube (PMT). The presence of the SHG signal is confirmed using the power dependence plots showing a slope of 2.06 in log-log scale and by acquiring the SHG spectrum (shown in the Supplementary Material, Figure S7). Polarization-dependent SHG (PSHG) measurements performed at the locations of interest are used to determine the incident light polarization that maximizes the SHG signal, thereby ensuring consistent comparison across the three samples irrespective of the flake orientation. Figure 3(d)–(f) shows the SHG images of the three samples, with the SHG signal strength for samples S2 and S3 at the location of interest being 57.6 and 54.6-times higher in comparison to sample S1 which is used here as the reference sample. Figure 3(g)–(i) shows the SHG images of the three samples with ∼270 nm PMMA encapsulation, with ∼2.2 and 2-times increase in SHG signal for samples S2 and S3, respectively. Figure 3(j) shows a comparison of the experimentally obtained (right axis) and simulated (left axis) SHG signals as a function of varying PMMA encapsulation layer thickness, showing good agreement. The SHG enhancements factors obtained from the experiments and simulations are included in the same plot. Overall SHG enhancement of ∼128 and 109-times are obtained for the PMMA encapsulated FP structures of samples S2 and S3 when compared to the reference sample S1.

**Figure 4:**
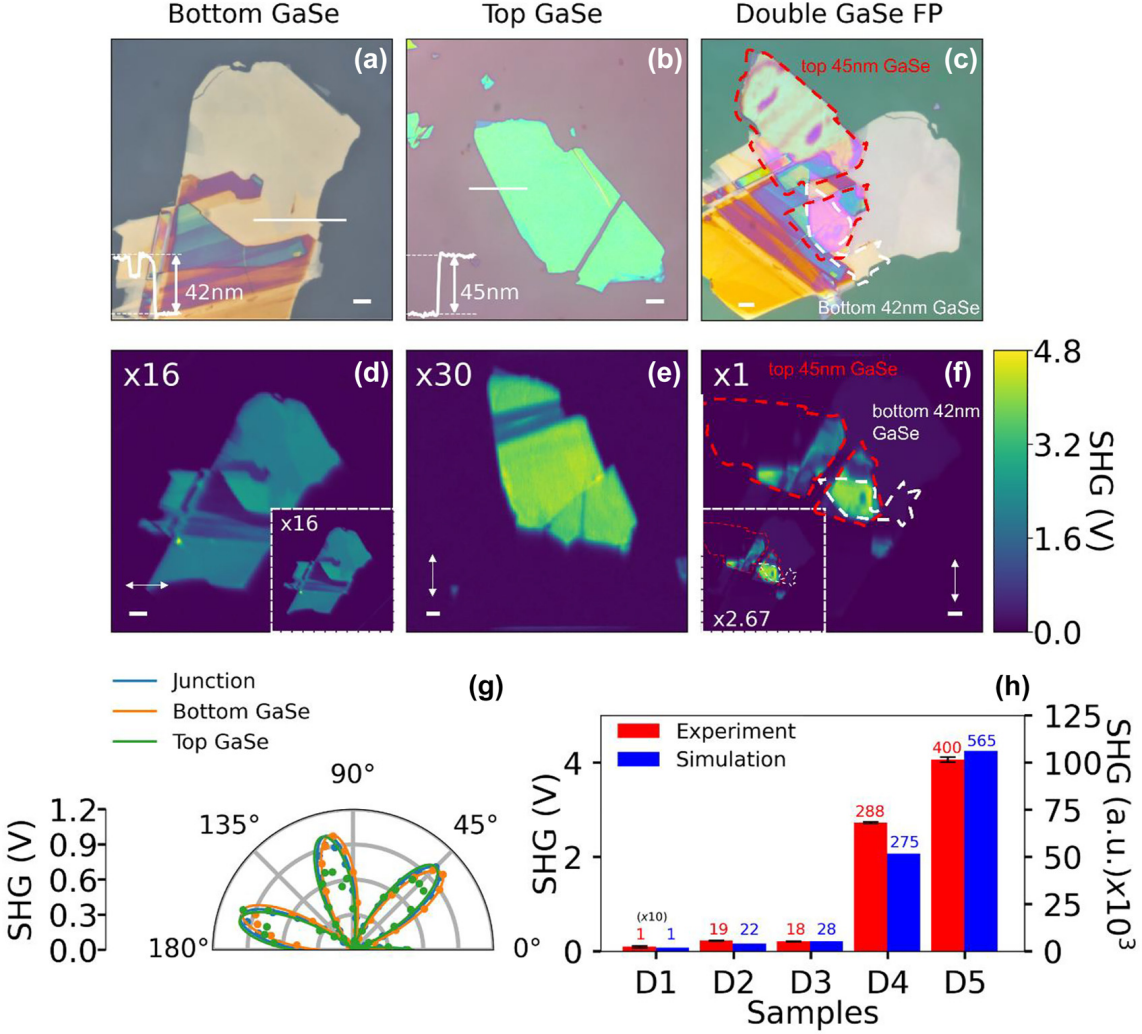
Summary of the experimental studies on the double GaSe FP structure. Optical images of samples: (a) 42 nm bottom GaSe/130 nm SiO_2_ without PMMA coating, (b) 45 nm top GaSe on PMMA coated substrate and (c) 270 nm PMMA encapsulated double GaSe FP structure. The red and white dashed outline highlights the region of interest for the top GaSe and bottom GaSe. SHG images of: (d) 190 nm PMMA spacer coated 42 nm bottom GaSe/130 nm SiO_2_, (e) 45 nm top GaSe on PDMS sheet and (f) 270 nm PMMA encapsulated double GaSe FP structure with the dashed outline delineating the top and bottom GaSe. Insets in (d, f) show the SHG image of the double GaSe FP structure without the PMMA layer. The armchair direction for both the top and bottom GaSe layers is indicated by the double-sided arrow. Scale bar for all optical and SHG image correspond to 5 µm. (g) Polar plot showing PSHG data correspond to top GaSe (green curve), bottom GaSe (orange curve) and the overlap region (blue curve) as a function of incident fundamental polarization angle. The circles show the experimental data and solid curves show the cos^2^(3*θ*) fit. (h) Bar-graph comparing experimental (red bars) and simulated (blue bars) SHG signal strength at different stages of fabrication of the double GaSe FP structure. The samples considered for comparison are: D1 (reference sample): 40 nm GaSe/300 nm SiO_2_, D2: 42 nm bottom GaSe/130 nm SiO_2_, D3: 190 nm PMMA/42 nm bottom GaSe/130 nm SiO_2_, D4: 45 nm top GaSe/190 nm PMMA/42 nm bottom GaSe/130 nm SiO_2_, and D5: 270 nm PMMA/45 nm top GaSe/190 nm PMMA/42 nm bottom GaSe/130 nm SiO_2_. The SHG enhancements factors for experiments and simulations are indicated on top of each bar graph.

Figure 4 summarizes the experimental results of SHG measurements performed at different stages of sample preparation for the double GaSe FP structure. The preparation of the double GaSe FP structures is discussed in detail in the methods section. Figure 4(a) shows the optical image of the bottom GaSe layer (∼42 nm thickness) on top of a 130 nm SiO_2_ layer deposited on a Si substrate. Figure 4(b) shows the optical image of the top GaSe layer (∼45 nm thickness) on a separate PMMA coated SiO_2_/Si substrate before being transferred onto a PDMS template for use as the top GaSe layer. Figure 4(c) shows the optical image of the final multilayer stack of the PMMA encapsulated double GaSe FP structure with the red and white dashed outlines showing the top and bottom GaSe layers, respectively. The overlap region of the red and white dashed region represents the complete multilayer stack with the thickness of the individual layers closely matching the HGA designed stack. Figure 4(d)–(f) shows the corresponding SHG images of the bottom GaSe layer in the presence of the 190 nm PMMA spacer layer, as prepared top GaSe layer and the final stack with 270 nm thick PMMA encapsulation layer, respectively. The samples exhibit reduced SHG signal strength before coating with PMMA spacer and encapsulation layers, respectively, as shown in the inset of Figure 4(d) and (f). For precise alignment of the top and bottom GaSe flakes, PSHG studies are performed separately on the top and bottom GaSe flakes to identify their respective armchair directions by rotating the sample while keeping the incident fundamental and analyzer orientations parallel to each other. The six-fold symmetric PSHG plots are used to guide the alignment of the two GaSe flakes using an optical microscope before the transfer process. Figure 4(g) shows the PSHG polar plots for the bottom GaSe (green data points), top GaSe (orange data points) and the overlap regions (blue data points). The relative twist angle between the top and bottom GaSe flakes after alignment is estimated to be ∼4°. Figure 4(h) shows a bar-graph comparing the experimental and simulated SHG enhancements for the double GaSe FP structure with sample D1 referring to the reference sample (40 nm GaSe/300 nm SiO_2_) and samples D2 to D5 being the multilayer stack at different stages of sample preparation, as indicated in the figure caption. The SHG enhancements factors obtained from the experiments and simulations are included on top of each bar graph. The experimental SHG enhancement shows good agreement with the trend observed in the simulations with a maximum enhancement of ∼400-times for the final stack (sample D5) when compared to sample D1. The small deviation observed in the SHG enhancement between the experiments and simulations is attributed to the differences in the measured PMMA spacer/encapsulation layer thickness and optical properties of the individual layers.

The highest normalized SHG conversion efficiency for the single and double GaSe FP structure, defined here as the ratio of the average SHG power to the square of the fundamental power $\left(\right.\eta_{2\omega }=P_{2\omega }/P_{\omega }^{2}$ ) as measured at the sample plane is calculated to be: *η*_2 ***ω***_ = **4.5** **× 10^−4^**/W and *η*_2 ***ω***_ = **1.43 × 10^−3^**/W, respectively. A comparison of the SHG conversion efficiencies obtained here with that of previous reports is summarized in Table T4 of the Supplementary Material. Resonant SHG enhancements that are an order of magnitude higher than the present report have been reported previously. However, the SHG conversion efficiency obtained here exceeds the values reported on 2D materials integrated with resonant metasurfaces or Bragg reflector cavities [22, 25, 26] by 1–2 orders of magnitude, with a much more simplified multilayer stack geometry. The improved SHG conversion efficiency obtained here is attributed to the design of the stack guided by the HGA routine with the objective of maximizing the SHG signal at the detector plane rather than only designing resonant structures for enhancing the fundamental field. The present dielectric stacks also offer a simpler light out-coupling geometry and are not prone to higher order diffraction effects at the SHG wavelength from the underlying periodic lattice, thus achieving higher overall SHG collection at the detector.

## 4 Discussion

**Figure 5:**
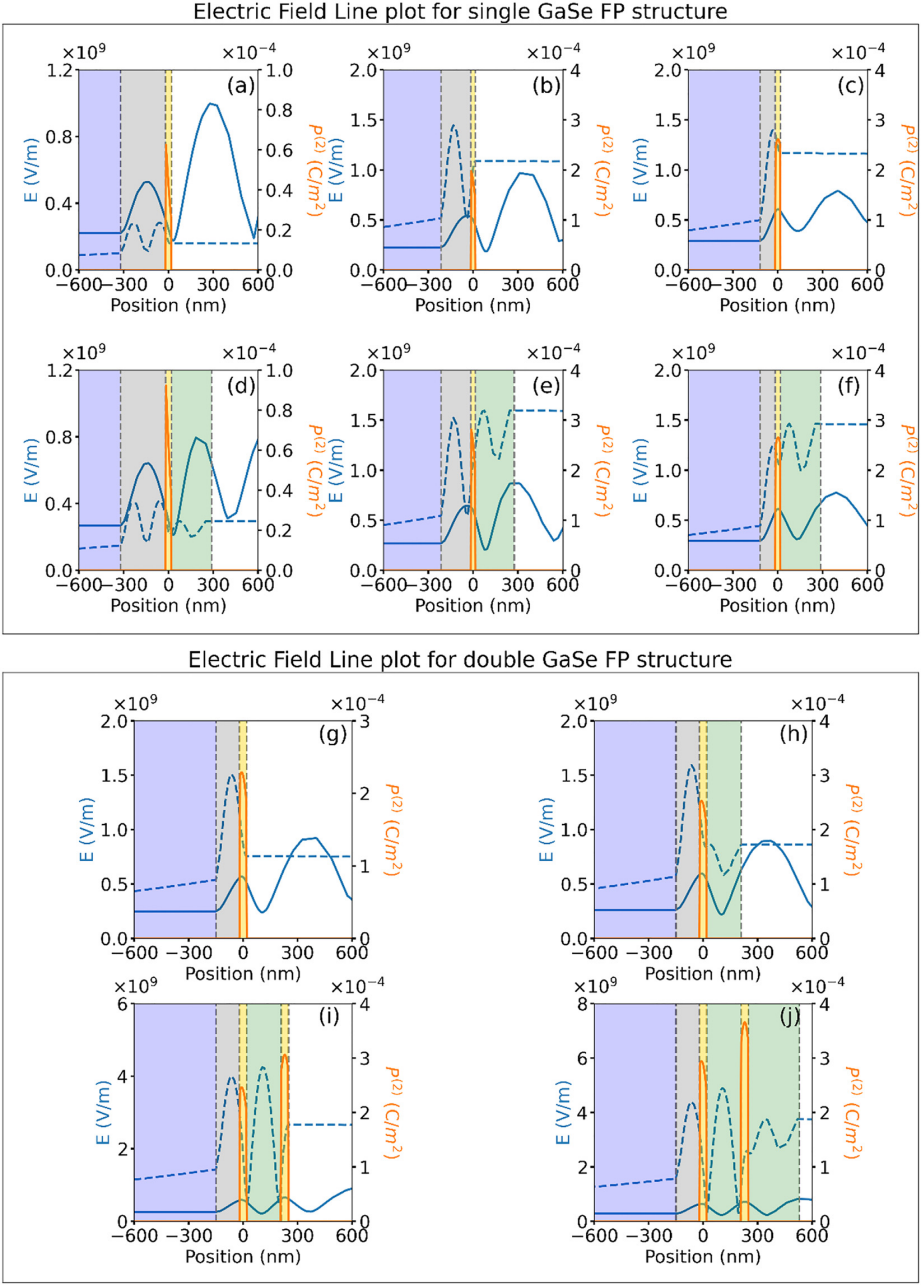
Simulated electric field line profiles at the fundamental (blue solid curves) and SHG (blue dashed curves, multiplied by 500) wavelengths and SHG non-linear polarization (orange solid curves) for single GaSe FP structure for the different samples fabricated with multilayer GaSe/SiO_2_ thickness values given as: (a, d) 35 nm/300 nm, (b, e) 30 nm/200 nm and (c, f) 35 nm/100 nm. The simulation results are shown without PMMA encapsulation layer (a–c) and with PMMA encapsulation layer (d–f). Simulated electric field line profile at the fundamental (blue solid curves) and SHG (blue dashed curves, multiplied by 500) wavelength and the SHG non-linear polarization (orange solid curves) plotted for double GaSe FP structure. The various multilayer stacks considered are: (g) 40 nm GaSe/130 nm SiO_2_, (h) 190 nm PMMA/40 nm GaSe/130 nm SiO_2_, (i) 40 nm GaSe/190 nm PMMA/40 nm GaSe/130 nm SiO_2_, and (j) 280 nm PMMA/40 nm GaSe/190 nm PMMA/40 nm GaSe/130 nm SiO_2_. Different layers in the stack are shaded for clarity as follows: Si (blue), SiO_2_ (grey), GaSe (yellow), PMMA (green), air region (white).

To get an intuitive understanding of the reason for the increased SHG from the HGA optimized FP structures, we compare the electric field and nonlinear polarization profiles across the multilayer stack. Figure 5(a)–(f) shows the simulated fundamental field (solid blue curves – left axis), SHG field (dashed blue curves – left axis) and SHG nonlinear polarization (red curves – right axis) line profiles for the optimized single GaSe FP structures with GaSe/SiO2 thickness of 35 nm/300 nm, 30 nm/200 nm and 35 nm/100 nm, both in the absence and presence of the PMMA encapsulation, respectively. The first-order standing-wave field pattern within the FP structure at the incident fundamental wavelength, ***λ*** satisfies the following condition: $\left(n_{SiO2}t_{SiO2}+n_{GaSe}t_{GaSe}+n_{PMMA}t_{PMMA}\right)=\frac{\lambda }{2}$ , where *n* and *t* are the refractive index and thickness of the individual layers, respectively. The multilayer stack studied here does not support high quality factor resonances due to the lack of high reflectivity mirrors or Bragg reflectors. Nonetheless, with the decrease in SiO_2_ layer thickness, increased overlap of the fundamental field with the GaSe layer is observed (comparing Figure 5(a)–(c)). The 100 nm SiO_2_ structure shows the peak of the fundamental field overlapping with the GaSe layer resulting in the highest SHG nonlinear polarization among the three samples. The SHG field build-up due to the coupling of the nonlinear polarization within the FP structure is found to result in the SHG field oscillating at approximately half the period of the fundamental wave. The addition of the PMMA encapsulation layer further increases the field build-up within the FP structure and corresponding out-coupling of the SHG field into the air region. The SHG field strength in the air region are found to be comparable for both 200 nm and 100 nm bottom SiO_2_ optimized structures (Figure 5(e) and (f)), resulting in comparable SHG strength obtained at the detector plane.

Similar observation can also be made for the double GaSe FP structures for which the optimized SiO_2_ layer thickness ensures good overlap of the peak of fundamental electric field with the bottom GaSe layer resulting in increased SHG nonlinear polarization (Figure 5(g)), which further increases in the presence of the PMMA spacer layer (Figure 5(h)). The addition of the top GaSe layer above the optimized thickness of PMMA spacer results in the two adjacent peaks of the fundamental field profile overlapping with the two GaSe layers resulting in an overall increase in SHG nonlinear polarization (Figure 5(i)). The top PMMA encapsulation layer further improves the SHG field coupled out of the FP structure into the air region when compared to the reference sample, resulting in multi-fold increase in SHG signal at the detector plane (Figure 5(j)). In summary, the improved overlap of the fundamental field with the GaSe layers, the corresponding higher SHG nonlinear polarization and increased SHG field strength in the presence of the PMMA encapsulation layer are found to result in overall improvement in the far-field collected SHG signal for the optimized designs.

It is also instructive to compare the SHG signal strength for the optimized double GaSe FP structure with a single GaSe layer of thickness equal to that of the sum of the two layers. A comparison of the simulated SHG signals for the reference sample, single 80 nm GaSe layer on 130 nm SiO_2_ and optimized double GaSe FP structure are shown in Figure S3 of the Supplementary Material. The optimized GaSe structure is found to increase the SHG signal by ∼160-times when compared to the single 80 nm GaSe layer on 130 nm SiO_2_. This improvement in overall SHG is attributed to the increased nonlinear polarization for the staggered structure due to good overlap with the fundamental field maxima.

Increased harmonic generation has been previously observed from monolayer, and few-layer TMDCs transferred onto polymer layers and flexible substrates due to strain relaxation effect [41, 42]. We have performed Raman spectroscopy and polarization dependent SHG studies to ascertain the role of strain relaxation in our study. Two different multilayer GaSe flakes of 40–45 nm thickness transferred onto ∼275 nm SiO_2_ layer and onto ∼280 nm PMMA layer on Si substrates are used for this study, as shown in Figure S8 of the Supplementary Material. The prominent Raman peaks from GaSe at 133.52 cm^−1^ (A^1^_1g_ mode), 212.6 cm^−1^ (E^1^_1g_ mode) and 307.97 cm^−1^ (A^2^_1g_ mode) are found to remain unchanged for the two samples. It is pointed out that previous report on Raman scattering studies on multilayer GaSe in the presence of intentional mechanical strain have shown prominent peak shifts and smearing [43] even for strain as low as 0.1%, with well-defined peaks observed for the as-transferred flakes similar to the Raman spectra obtained in the present study. We also find that the PSHG study shows clear six-fold symmetry which fits very well with a cos^2^(3*θ*) dependence (refer to Figure 4(g) and Figure S8 of the Supplementary Material). The effect of strain on the SHG measurements is often seen as a clear asymmetry in the six fold PSHG plot [44], which is clearly absent in the present study, thus eliminating any strain and subsequent relaxation effects associated with the samples studied here. Hence, the negligible peak shifts/broadening observed in the Raman spectra, the six-fold symmetric PSHG data and good agreement between the experiments and nonlinear wave-propagation simulations without considering strain effects leads to the conclusion that strain relaxation effects play negligible role in enhancing SHG in the multilayer FP structures.

## 5 Conclusions

Here we have reported a hybrid genetic optimization algorithm for designing multilayer GaSe FP structures for SHG enhancement studies. The design and experimental studies underscore the usefulness of genetic optimization to accelerate the design of 2D material-based FP structures when compared to the “brute force” multi-parameter sweeps, which requires significantly higher computational resource. The HGA technique utilized in this work speeds up the design process by ∼8.8 and 89-times for the single and double GaSe FP structures, respectively, when compared to the full parameter-sweep approach, considering sequential execution of the electromagnetic simulations. Further speed-up is expected through parallel execution of the simulations and using previous runs to guide the genetic evolution. The experimental measurements show an overall SHG enhancement of ∼128 and 400-times for the HGA designed single and double GaSe FP structures, respectively, with good agreement with the simulation studies. A closer look at the field and polarization profiles shows that the optimized multilayer FP design with PMMA encapsulation improves the far-field SHG detection by ensuring good overlap of the fundamental field with the nonlinear medium and at the same time having good SHG field build-up and out-coupling from the structure. The multilayer stack fabrication in the present work is restricted to micron-sized area due to the dry-transfer technique, however there is good potential for scaling up to larger areas using advances in 2D material deposition techniques [45]. The present work is focused on HGA designs with two separate multilayers 2D materials with dielectric interlayers. Nonetheless, the optimization algorithm utilized here is robust and can easily be extended to nonlinear optical enhancement studies of more complex multilayer stacks of dissimilar 2D materials with non-zero twist angles [4] and heterogeneously integrated 2D material – resonant photonic structures [28].

## 6 Methods

**Hybrid genetic optimization algorithm (HGA):** The HGA utilized here is a numerical evolutionary optimization technique combining genetic algorithm (GA) with stochastic hill climbing (SHC) for fast convergence to the optimum solution. A flow-chart of the HGA is shown in Figure 1(c). The algorithms start with an initialization step by selecting a randomly initialized population. The population is composed of a fixed number of individuals, with each individual representing a specific multilayer stack. The thickness of each layer for a multilayer stack is chosen within a range of values, as listed in Figures 2(b)–(d) and 4(b)–(e). The thickness parameters are binary encoded within the HGA implementation. The SHG signal strength is calculated for each individual using nonlinear-optical wave propagation simulation implemented using COMSOL, as discussed below. The individuals are sorted based on the SHG signal strength and a pre-determined number, 20 (40) for the single (double) GaSe FP structure is selected for generating the next generation of individuals through proportionate selection, crossover, and mutation operation. Proportionate selection, also termed as roulette wheel selection is used to associate fitness ranking with SHG values of multilayer stack. Individuals with higher fitness have higher probability for selection for creating the next generation of population. Next, single point crossover is applied where a single point is arbitrarily chosen beyond which the bit string is swapped among two individuals. Subsequently, single bit flip through the process of mutation is applied to an arbitrary bit of the individual with a 10% probability to ensure diversity in the population.

After multiple iteration of GA, the solution is said to have temporarily converged when the results remain unchanged for 20 successive iterations. The individual with the highest SHG value is used as the starting point for the SHC algorithm. The SHC algorithm compares the SHG value for the identified starting point with that of neighbouring individuals which are defined as multilayer stacks with a single step size difference in any one of the thickness parameters with respect to the starting stack. If the SHG value of neighbouring stack is higher than the starting point, then the thickness parameters are updated with the improved neighbouring stack parameters. Since the SHC algorithm requires more data points to be simulated, the algorithm is used only once. Subsequently, the HGA reverts to GA for further exploring the parameter space for better solutions. This process continues till the best possible solution is identified, when the solution remains unchanged for 50 consecutive iterations. HGA is implemented in MATLAB [46] in combination with COMSOL through Livelink for MATLAB module [47]. The settings used in the HGA are tabulated in Table T1 of the Supplementary Material.

**Nonlinear wave propagation simulation:** To model the fundamental excitation source and the SHG emission from the multilayer stack, a two-dimensional (2D) model is defined in COMSOL multiphysics in the wave-optics module [47]. The cross-section of the multilayer stack as defined in COMSOL is shown in Figure S1 of the Supplementary Material. The complex refractive index for each layer used in the simulation for the wavelengths of interest is listed in Table T2 in the Supplementary Material. The 2D electromagnetic simulations are found to be a good approximation for the full three-dimensional simulation with considerably reduced computational time. An input Gaussian beam with central wavelength of 1040 nm is used as the fundamental excitation with the spot diameter and peak electric field values defined based on experimental measurements. The fundamental excitation results in a spatially varying electric field across the multilayer stack, which is denoted by $\vec{E_{1}}$ . The in-homogeneous wave equation used to calculate the corresponding SHG electric field, $\vec{E_{2}}$ within the multilayer FP structure as a function of the second order nonlinear polarization, $\vec{P_{2}^{\left(2\right)}}$ is given as follows [40]:

$$\nabla^{2}\vec{E_{2}}-\frac{\varepsilon \left(2\omega \right)}{c^{2}}\left(\frac{\partial^{2}\vec{E_{2}}}{\partial t^{2}}\right)=\frac{1}{\varepsilon_{0}c^{2}}\left(\frac{\partial^{2}\vec{P_{2}^{\left(2\right)}}}{\partial t}\right)$$

The time-dependent SHG nonlinear polarization that acts as the driving term for the SHG process is given as: $P_{2i}^{\left(2\right)}=\varepsilon_{0}\chi_{ijk}^{\left(2\right)}E_{1j}E_{1k}$ , with *i*, *j*, *k* being the directional indices and $\chi_{ijk}^{\left(2\right)}$ , the second-order nonlinear susceptibility at the SHG wavelength. The wave propagation equation for the SHG field is solved using finite element method (FEM) in COMSOL. The SHG signal strength is calculated in the backward direction i.e., in the air region above the PMMA encapsulation layer by taking a line integral of the pointing vector at the SHG wavelength centered at 520 nm wavelength at the 1D detector placed 1.2 μm from the PMMA layer.

**Sample preparation:** For the fabrication of a single GaSe FP structures, three different samples with varying SiO_2_ thicknesses of 300 nm, 200 nm and 100 nm denoted as S1, S2 and S3, respectively are deposited on a Si wafer using plasma-enhanced chemical vapor deposition (PECVD) process. GaSe material obtained from 2D Semiconductor Inc. is exfoliated and transferred onto the three samples using standard dry-transfer method. The dry transfer setup consists of a zoom lens system, XY sample stage with a rotational mount and a separate XYZ stamp stage [48]. The thickness of GaSe close to the HGA determined values is identified using optical and AFM imaging, as shown in Figure S6 of the Supplementary Material. For the top encapsulation layer, polymethyl methacrylate (PMMA-A4) layer is spin coated on top of the samples at a spin speed of 2200 rpm, resulting in PMMA layer thickness of 270 nm.

For the fabrication of double GaSe FP structures, the process flow is divided into two parts to ensure angle aligned transfer of top GaSe flake on top of the bottom GaSe layer. First, the bottom SiO_2_ layer of thickness 130 nm is deposited on a Si wafer using PECVD. The bottom multilayer GaSe exfoliated using dry-transfer method is transferred on top of the SiO_2_ layer, with thickness of ∼42 nm identified using AFM imaging. The sample is subsequently baked for 2 min at 180 °C temperature to remove any interfacial bubbles and to promote adhesion. PMMA-A4 is subsequently spin-coated on the sample at a spin speed of 5000 rpm to achieve 190 nm PMMA spacer layer. Next, the top GaSe flake is prepared using a PMMA assisted transfer method, where the second multilayer GaSe flake is exfoliated and transferred onto a separate PMMA coated SiO_2_-Si substrate to identify ∼45 nm thick GaSe using AFM imaging. This step is required due to the large surface roughness of the PDMS sheet which prevented accurate AFM measurement. Subsequently, the GaSe sample is covered with a polydimethylsiloxane (PDMS) sheet and immersed in acetone in an inverted set-up with a linear stage used for vertical manipulation. Once the PMMA layer is completely dissolved in acetone, the GaSe flake attaches to the PDMS sheet, and is used as the top GaSe layer. PSHG measurements are performed separately on the bottom and top GaSe samples to determine the armchair axes. The two samples are subsequently brought together in a dry-transfer setup for angle aligned transfer. By comparing optical image of the two samples, the bottom GaSe is rotated relative to the top GaSe with the angular offset determined using PSHG measurements. After angular alignment, the top GaSe is transferred above the PMMA coated bottom GaSe sample and baked for 2 min at 180 °C to promote adhesion. A 270 nm thick PMMA encapsulation layer is subsequently spin coated at a speed of 2200 rpm followed by a final baking step. The PSHG measurements on the final multilayer stack showed that the angular mismatch between the top and bottom GaSe layers is ∼4°, as seen in Figure 5(g). High quality optical image for both single and double GaSe FP structure are obtained using an optical microscope (Leica DM2500).

**Nonlinear microscopy system for SHG studies:** We use a standard nonlinear microscopy setup for the SHG measurement as show in the schematic in Figure S10 of the Supplementary Material. A 1040 nm fs laser source (Fidelity-HP) with a pulse duration 140 fs and repetition rate of 80 MHz is used as a fundamental excitation source. A half wave plate and polarizer are used to control the power and set the initial polarization state of the incident light source. The fundamental excitation source with a maximum average power of 0.6 mW is focussed on the sample using a 20×/0.75 NA objective lens. The focal spot diameter of the incident fundamental is estimated to be 2.04 μm. The device under test is mounted on the stage of an inverted microscope (Olympus, IX73). Backward propagating SHG is collected in the epi-detection geometry using the same objective and directed to a photo-multiplier tube (PMT) using a dichroic beam splitter. A combination of band-pass (520 ± 40 nm and 520 ± 15 nm) and short-pass (one 890 nm short pass) filters are used to reject the residual pump and any background signal in the detection path with ∼150 dB extinction to spectrally separate and detect only the SHG signal. A pair of galvanometric mirrors is used to scan the fundamental excitation source onto the sample and map the PMT signal to form SHG images. The incident power dependence of the collected SHG signal shows a quadratic relationship, as seen in the log-log plot in Figure S7 of the Supplementary Material with a slope of ∼2, confirming the second-order nature of the nonlinear optical process. A representative SHG spectra for the single GaSe FP structure acquired using a spectrometer (Andor Kymera 328i) coupled to an EMCCD camera (Andor iXon Ultra 897 BVF) is also shown in Figure S7 of the Supplementary Material. The experimental SHG data from the three samples shown in this plot are normalized with respect to that of a standard quartz window, measured in the same run. This ratiometric analysis eliminated any variability in experimental conditions for the different measurement runs.

For PSHG study, we utilize two different techniques for single and double GaSe FP sample. For single GaSe samples, the sample is placed on a glass slide in a fixed position on the microscope. The input polarization of the laser source is rotated over 0 to 360° in steps of 1° using a half wave plate placed in between the input laser source and the microscope, with an analyzer placed in front of PMT aligned parallel to the laboratory horizontal axis (*x*-axis). This measurement resulted in four-fold symmetric SHG polar plot [49], as shown in Figure S7 of the Supplementary Material. This technique is simpler to implement and is sufficient for determining the incident polarization direction that maximizes SHG signal. For the double GaSe FP stacks, the sample is placed on a rotatory mount which is rotated from 0 to 180° in steps of 5°. The input polarizer and the output analyzer are kept fixed and parallel to the laboratory horizontal axis (*x*-axis). This results in six-fold symmetric SHG polar plot [2], which is used to determine the GaSe layers arm-chair axis to within ±0.5° accuracy. This measurement is used to guide the angle aligned transfer of top GaSe on the bottom layers, as discussed in the sample preparation method above.

For the SHG conversion efficiency calculations, the PMT voltage measured is converted to the SHG optical power at the sample plane by accounting for the objective lens collection efficiency in the backward geometry and any additional optical loss along the path. This is done by using the halogen lamp above the condenser in the inverted microscope as the light source which is focused on the sample plane with suitable aperture stop, collected by the objective lens and directed to the PMT. The PMT signal measured as a voltage, which is kept comparable to the SHG signal voltage in the SHG experiments is scaled to the optical power at the sample plane by measuring the power using a sensitive power-meter. For the SHG measurements on single (double) GaSe FP structures, the average incident fundamental power of 0.55 mW (0.275 mW) at the fundamental wavelength, which corresponds to peak optical intensity of 14.44 GW/cm^2^ (7.2 GW/cm^2^) resulted in average SHG optical power of 0.161 nW (0.108 nW). The normalized conversion efficiency from these measurements is estimated to be 4.5 × 10^−4^ /W (1.43 × 10^−3^ /W).

**Raman scattering Measurements:** Raman scattering measurements were performed using a micro-Raman spectrometer (LabRAM HR from Horiba) system equipped with grating of 1800 lines/mm and a Peltier-cooled CCD array (Syncerity). The Raman spectra are recorded at an optical resolution of 0.3 cm^−1^ by using a 532 nm laser excitation source focused on the sample using 100× objective.

## Supplementary Material

Supplementary Material Details
