# Supplementary material for: Evolutionary design of two-dimensional material Fabry–Perot structures for enhanced second harmonic generation
Source: Nanophotonics. 2022 Dec 15;12(1):29–42. doi: 10.1515/nanoph-2022-0459 (PMC11501989; doi:10.1515/nanoph-2022-0459)
Supplement: Supplementary file 1 — Supplementary Material Details [file j_nanoph-2022-0459_suppl_001.docx]

**Supplementary Information**

**Evolutionary Design of Two-dimensional Material Fabry-Perot Structures for Enhanced Second Harmonic Generation**

Rabindra Biswas, Asish Prosad, Lal Krishna A. S, Sruti Menon, Varun Raghunathan*

Department of Electrical Communication Engineering,

Indian Institute of Science, Bangalore 560012, India

*varunr@iisc.ac.in

**Parameters used in hybrid genetic optimization algorithm**

Table T1 shows the set of parameters used in the implementation of hybrid genetic algorithm (HGA). The thickness of PMMA is limited by the thickness range possible as limited by the spin-speed for PMMA-A4 and A2 resist. For GaSe and SiO_2_, it has been observed in the trend of the thickness dependence SHG that SHG signal strength periodically repeats itself after certain thickness due to phase matching consideration[1]. The thickness range for GaSe and SiO_2_ are chosen considering the total device to be kept as thin as possible.

**Table T1: Parameters used in hybrid genetic optimization algorithm**

| **Population size** | **Elitism** | **Crossover** | **Mutation** | **Thickness parameters and range** | | |
| --- | --- | --- | --- | --- | --- | --- |
| 20 (Single GaSe),  40 (Double Cavity GaSe) | 25% | 80% | 10% | Materials | Thickness range | Step Size |
|  |  |  |  | PMMA | 100-400nm | 10nm |
|  |  |  |  | GaSe | 5-50nm | 5nm |
|  |  |  |  | SiO_2_ | 50-300nm | 10nm |

**Optical properties of material used in the design studies**

The complex refractive index (n,k) parameters used for SHG simulation in COMSOL simulation is summarized in the Table T2.

**Table T2: Complex refractive index of the materials used in the design studies**

| **Material** | **At fundamental wavelength (1040nm)** | **At SHG wavelength (520 nm)** | **References** |
| --- | --- | --- | --- |
| Silicon | 3.562,0 | 4.187,0.04 | Palik [2] |
| SiO_2_ | 1.4996,0 | 1.5021,0 | Measured using Ellipsometry |
| PMMA | 1.496,0 | 1.4837,0 | [3] |
| GaSe | 2.7991,0 | 3.0852,0.3263 | [4,5] |

**Table T3: Comparison of computation time for HGA and full-parameter space sweep**

| **FP structure considered** | **Hybrid Genetic Algorithm (HGA)** | | **Full parameter space sweep** | | **Magnitude of computational time improvement for HGA in comparison to full-parameter sweep** |
| --- | --- | --- | --- | --- | --- |
|  | **Number of objective function evaluated^#^** | **Time required for a full HGA run^#^*** | **Number of objective function evaluated** | **Time required for a full run*** |  |
| **Single GaSe FP Cavity** | 914 | 3.8 hrs | 8060 | 33.5 hrs | 8.8-times |
| **Double GaSe FP Cavity** | 2801 | 14 hrs | 249860 | 52 days | 89-times |

*****Single SHG simulation in a Intel Core-i5 processor, 46GB RAM takes around 15-18sec.

**^#^** The HGA settings as listed in table T1 above

The above comparison assumes sequential computation of each electromagnetic simulation. The use of parallel computation can speed up both HGA and full-parameter sweep. The HGA population can be run in parallel (20 for single GaSe FP structure and 40 for double GaSe FP structure). Furthermore, the prior HGA runs can be used to guide the subsequent runs using suitable look-up table to avoid repetition of parameter space search.

**Table T4: Comparison of the SHG conversion efficiencies calculated in this work with previous reports on 2D material integrated resonant photonic structures**

| **References**  **(as per main paper)** | **Description of the structure** | **Fundamental excitation wavelength** | **Enhancement factor** | **Normalized conversion efficiency** | **Other comments** |
| --- | --- | --- | --- | --- | --- |
| Ref. 17 | Few layer GaSe on photonic crystal cavity | 1528 nm | 1320 | Not reported | Continuous wave fundamental input used |
| Ref. 19 | Monolayer WS_2_ on circular bragg grating | 1233 nm | 5 | Not reported | -- |
| Ref. 20 | Monolayer MoS_2_ sandwiched between SiN/SiO_2_ DBR cavity | 818 nm | 10 | Not reported | -- |
| Ref. 21 | MoS2 flake with DBR bottom mirror and silver top mirror | 925 nm | 3300 | 1.68x10^-8^/W | -- |
| Ref. 23 | Multilayer MoS_2_ on SiO_2_-SnO_2_/Ag/SiO_2_ | 800 nm | 18 | Not reported | -- |
| Ref. 24 | Monolayer MoS_2_ on Ag film hole array | 868 nm | 1527 | Not reported | -- |
| Ref. 25 | Multilayer GaSe on quasi-bound state in continuum structures | 1543.9 nm | 26 | 2.477x10^-5^/W | Continuous wave fundamental input used |
| Ref. 26 | Multilayer GaSe on silicon nanodisk array | 1620 nm | 22 | 3.5x10^-6^/W | Polarization independent SHG enhancement |
| Ref. 28 | Multilayer GaSe on quasi-BIC metasurface | 1334 nm | 9400 | Not reported | Continuous wave fundamental input used |
| **This work** | **Single multilayer GaSe sandwiched between bottom SiO_2_ and PMMA encapsulation layer** | **1040 nm** | **128** | **4.5x10^-4^/W** | **Use of HGA to design the multilayer FP stack** |
| **This work** | **Double multilayer GaSe sandwiched between bottom SiO_2_, PMMA space and encapsulation layer** | **1040 nm** | **403** | **1.43x10^-3^/W** | **Use of HGA to design the multilayer FP stack** |


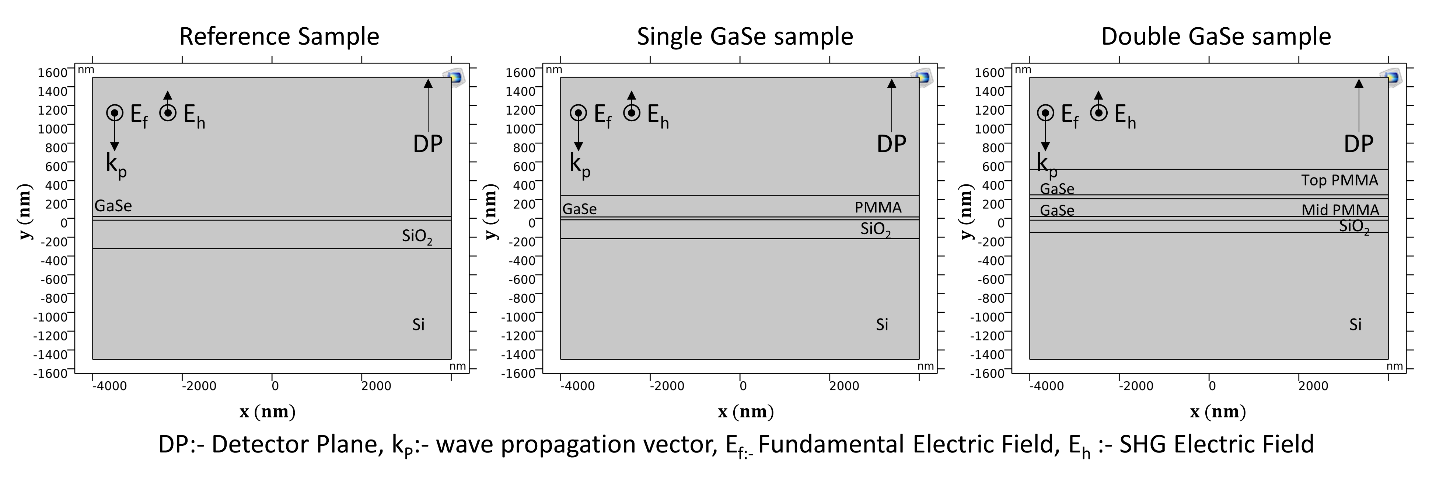


Figure S1: The electromagnetic simulation cross-section for :(a) reference sample of multi-layer GaSe on SiO_2_/ silicon substrate, (b) PMMA encapsulated multilayer GaSe on SiO_2_/ silicon substrate, and (c) two GaSe layers with PMMA spacer and encapsulation layer on SiO_2_/ silicon substrate.

**
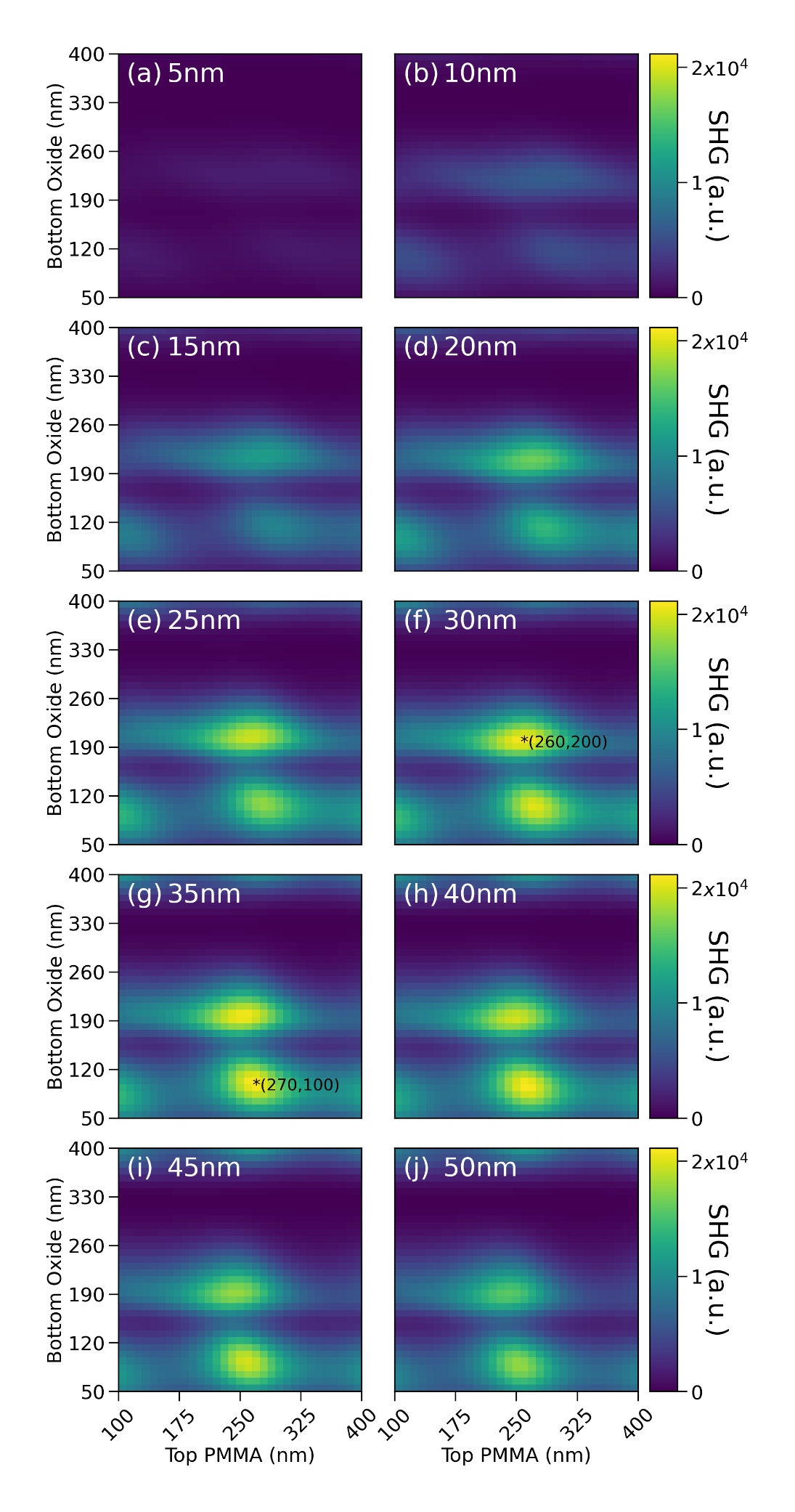
**

Figure S2: SHG contour map of single GaSe stack as function of top PMMA, bottom SiO_2_ and GaSe thickness shows the results of sequential search over the predefined thickness range. GaSe thickness is indicated in the top-left side of each contour plot. The two optimized designs (PMMA/GaSe/SiO_2_ of 260nm/30nm/200nm and 270nm/35nm/100nm) are marked by asterisks in the contour plot.


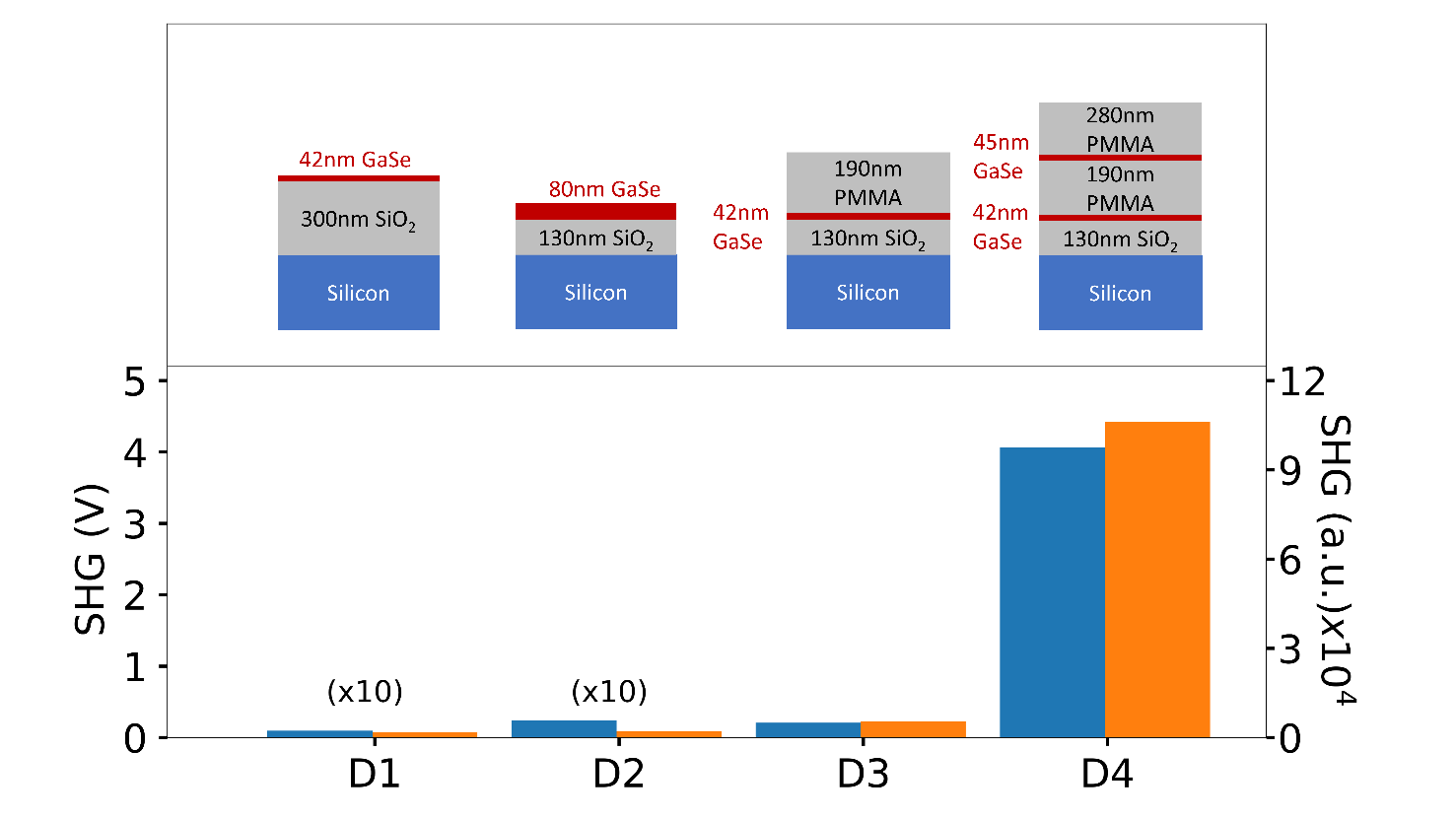


Figure S3: Comparison between the SHG of different multilayer stacks as shown above the bar chart. The simulation results (orange bar) and experimental results (blue bars) are shown. The SHG enhancement factors for the experiments (simulations) are: 1 (1) for 42 nm GaSe/300 nm SiO_2_, 2.536 (1.08) for 80 nm GaSe/130 nm SiO_2_, 21.9 (28.55) for 190 nm PMMA/42 nm GaSe/130 nm SiO_2_ and 429.6 (565.8) for 280 nm PMMA/45 nm GaSe/190 nm PMMA/42 nm GaSe/130 nm SiO_2_.

**Sensitivity Analysis of the HGA optimized designs**

A sensitivity analysis for the optimized designs is also performed to estimate the variation in SHG signal strength due to variation in individual layer thickness due to fabrication and measurement nonidealities. Based on ellipsometry and atomic force microscopy (AFM) measurements on uniform films and GaSe flakes respectively, we estimate the expected error in thickness of SiO2, PMMA and GaSe layers to be +/- 10 nm, +/- 10 nm and +/- 2 nm respectively. We calculate the SHG signal strength for the FP structures taking into consideration the above thickness variation around the optimized values. We find that for the case of the single (double) GaSe FP structure, the decrease in SHG signal is estimated to be within 85% (75%) of its maximum value for majority of the thickness variation considered here. A summary of the sensitivity analysis is shown as bar graphs in figures S4 and S5 for the single and double GaSe FP structures respectively.


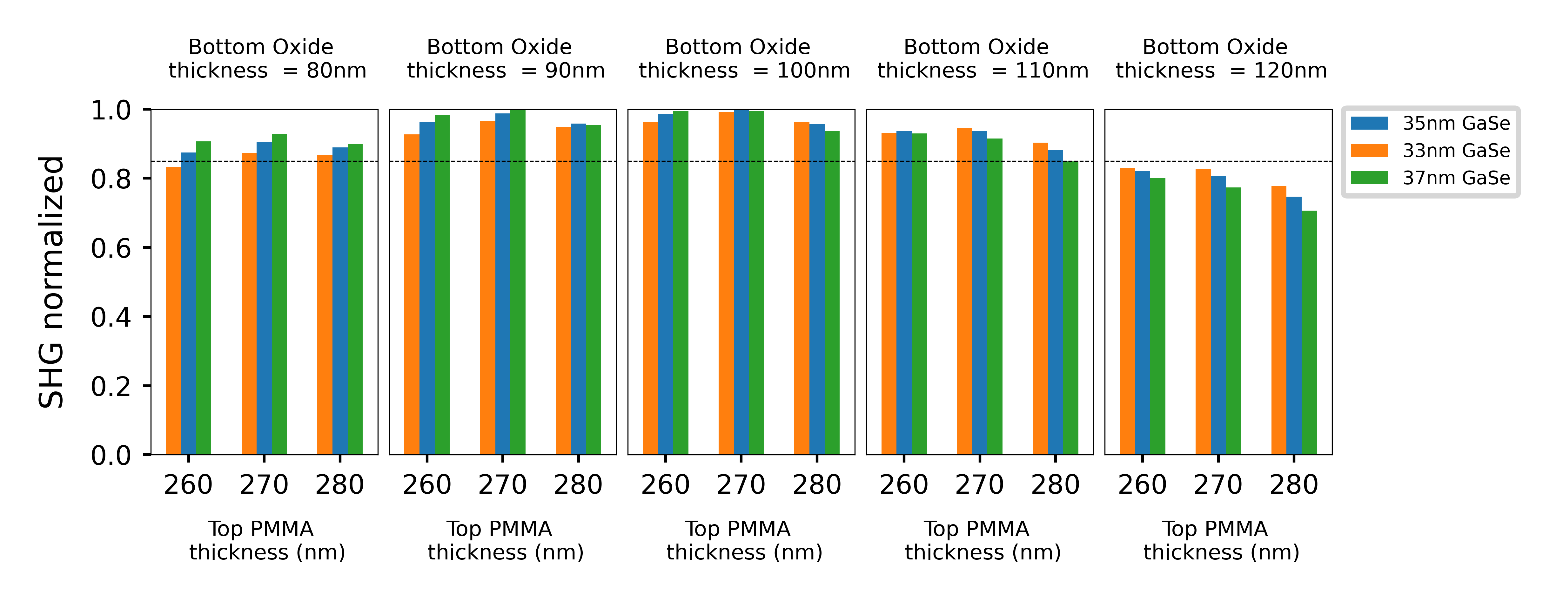


Figure S4: Sensitivity analysis for single GaSe FP structure showing the SHG signal as a function of variation in thickness of each individual layers (GaSe layer: 33 nm±2nm, bottom SiO_2_ layer: 100 nm±10nm and top PMMA layer: 270±10nm).


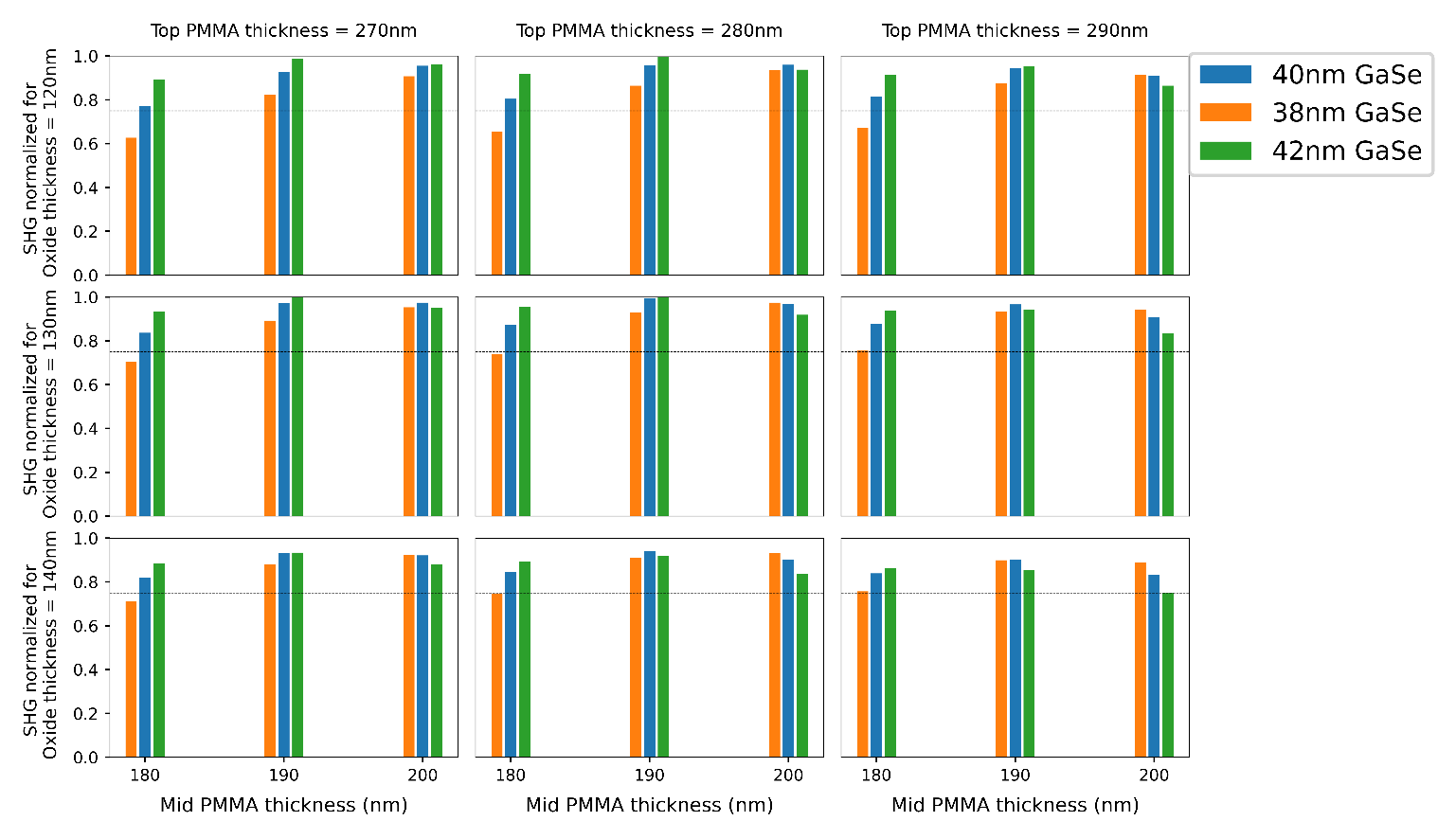


Figure S5: Sensitivity analysis for double GaSe FP structure showing the SHG signal as a function of variation of thickness in individual layers (GaSe layer: 40±2nm, bottom SiO_2_ layer: 130nm ±10nm, top PMMA layer: 280 nm ±10nm and mid PMMA layer: 190nm±10nm).

**
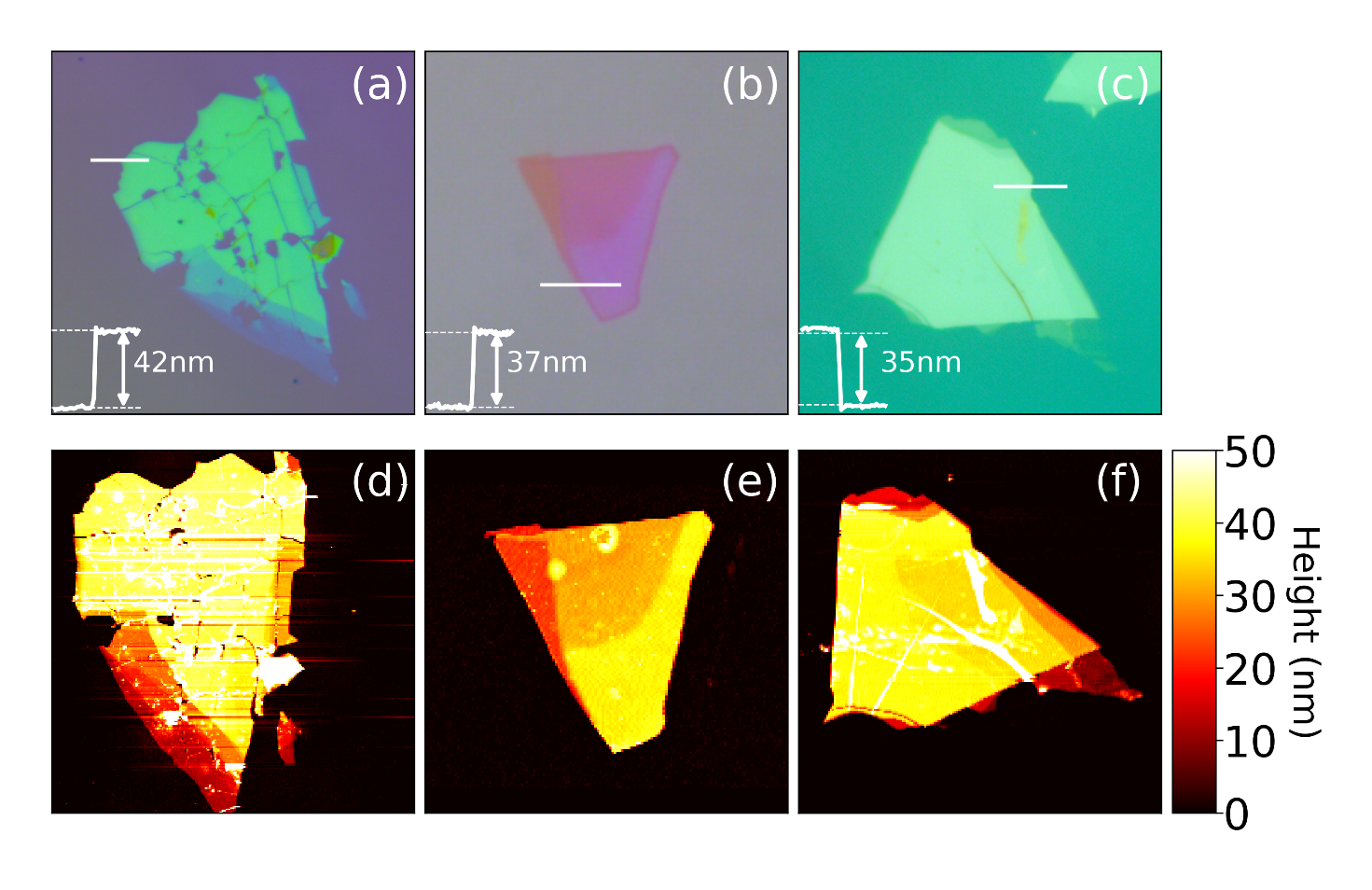
**

Figure S6: Optical and atomic force microscopy image of single GaSe FP structure samples on SiO_2_-silicon substrate for three different SiO_2_ thickness: (a,d) 300nm, (b,d) 200nm, and (c,f) 100nm. The inset in (a-c) shows the AFM line profile for the location marked by the white line.


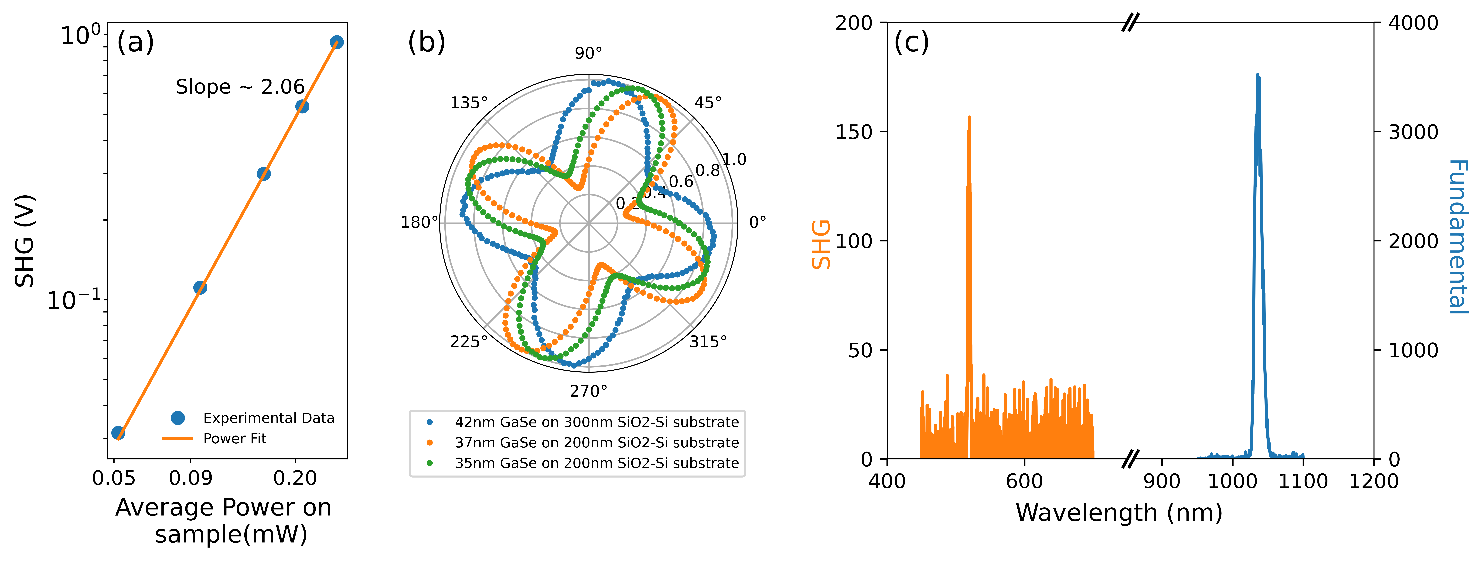


Figure S7: (a) Input power dependence of the SHG signal from GaSe shown as a log-log plot. (b) Polarization dependent SHG measurement on three single GaSe FP samples with different SiO_2_ thickness as indicated in the legend above. (c) Optical SHG spectrum of GaSe thin flake on SiO_2_-Silicon substrate


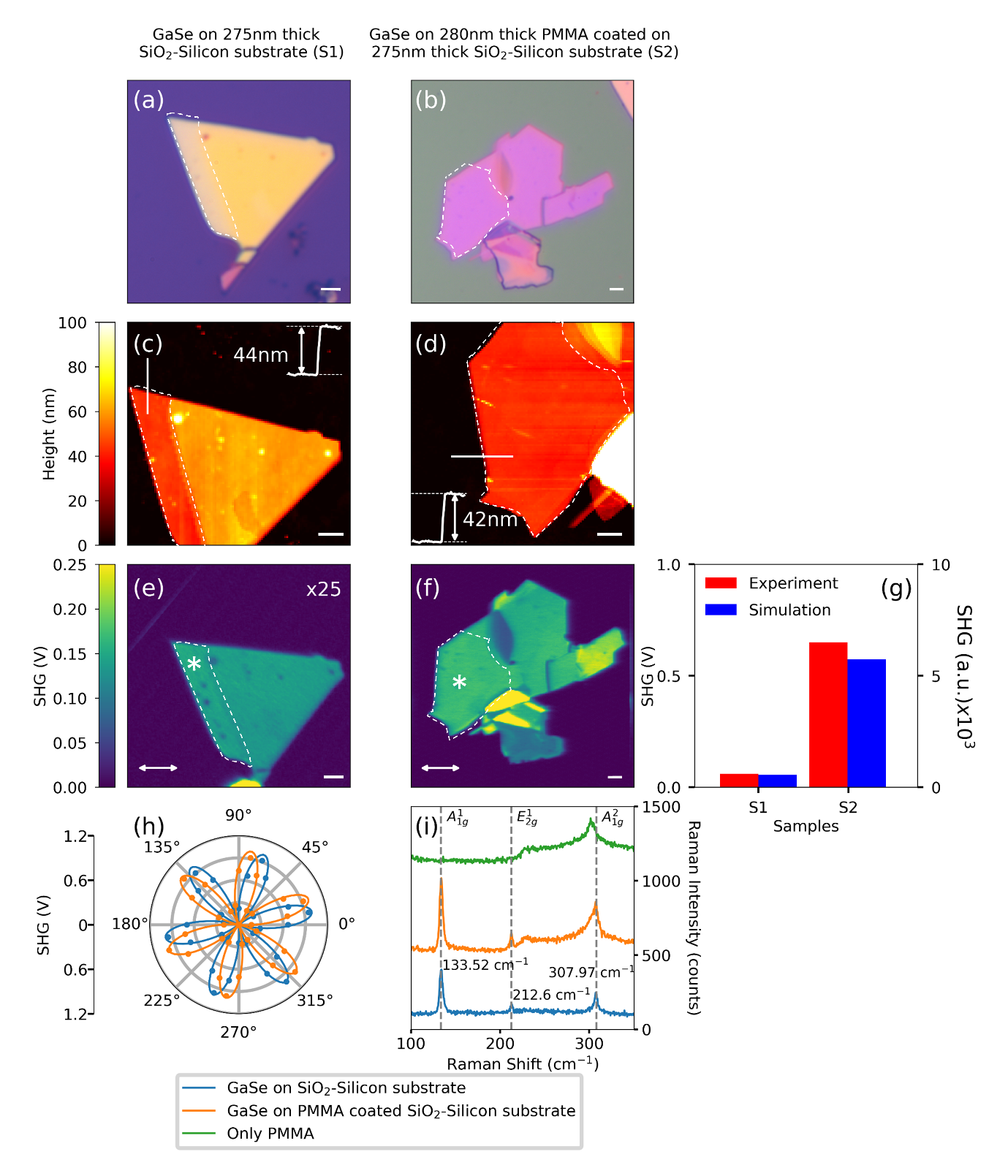


Figure S8: (a,b) Optical images, (c,d) atomic-force microscopy images, (e,f) SHG microscopy images for multilayer GaSe on 275 nm SiO2 layer/ Si substrate (left coloumn) and multilayer GaSe on 280 nm PMMA layer/ 275 nm SiO2 layer/ Si substrate. (g) Bar graph plot comparing the experimental (red) SHG data points with the SHG simulation (blue) SHG results for the two samples. (h) Polarization dependent SHG studies for the white dashed region. (i) Raman spectra for the two samples and comparison with PMMA layer.


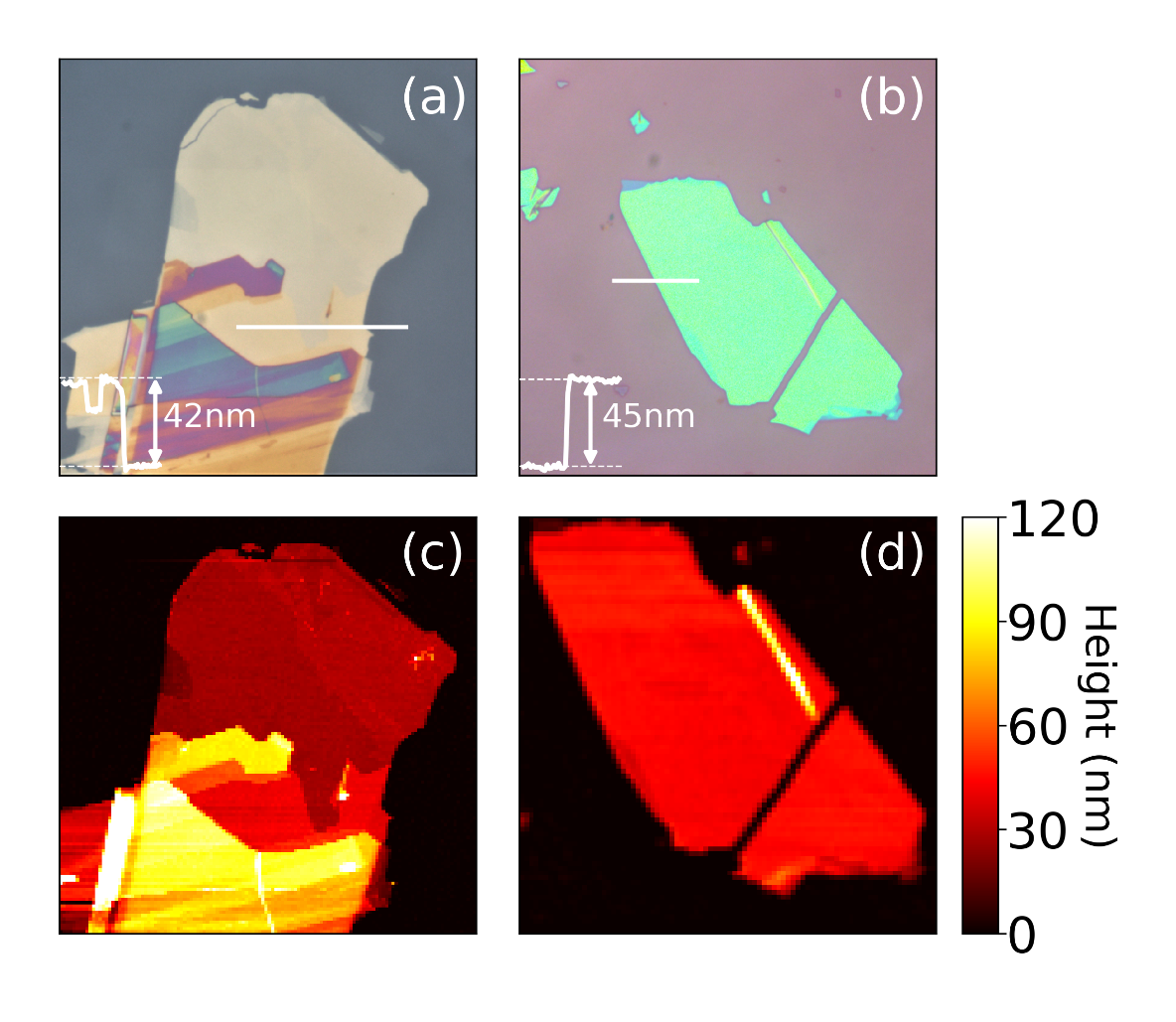


Figure S9: Optical and atomic force microscopy images of bottom and top GaSe flakes for double GaSe Fabry-Perot cavity (a,c) Bottom GaSe (b,d) Top GaSe. Thickness for region of interest is indicated by a line scan in the respective optical image.

Figure S10: Nonlinear Microscopy setup for SHG measurements in backward (reflection) mode.

**Reflection Measurements on the GaSe FP structures**

Reflection measurements were performed using the pulsed 1040 nm femtosecond laser source (Fidelity-HP) incident on the sample with the reflected light collected using the same focusing optics. The reflected beam is then separated from the incident beam using an AR coated 50:50 beam splitter. At the output of the microscope, the reflected light is coupled to a 50 μm multimode fiber using a fiber-port coupler (Thorlabs PAF2A-15B) to collect the reflected light and couple it to an optical spectrum analyzer (YOKOGAWA AQ6370D) to obtain the reflection spectrum. The reflectance plot is obtained by normalizing the reflection from the GaSe region with respect to a gold mirror placed at the sample plane. The comparison between the simulated and experimentally measured reflectance spectra are shown in figure S11 below.


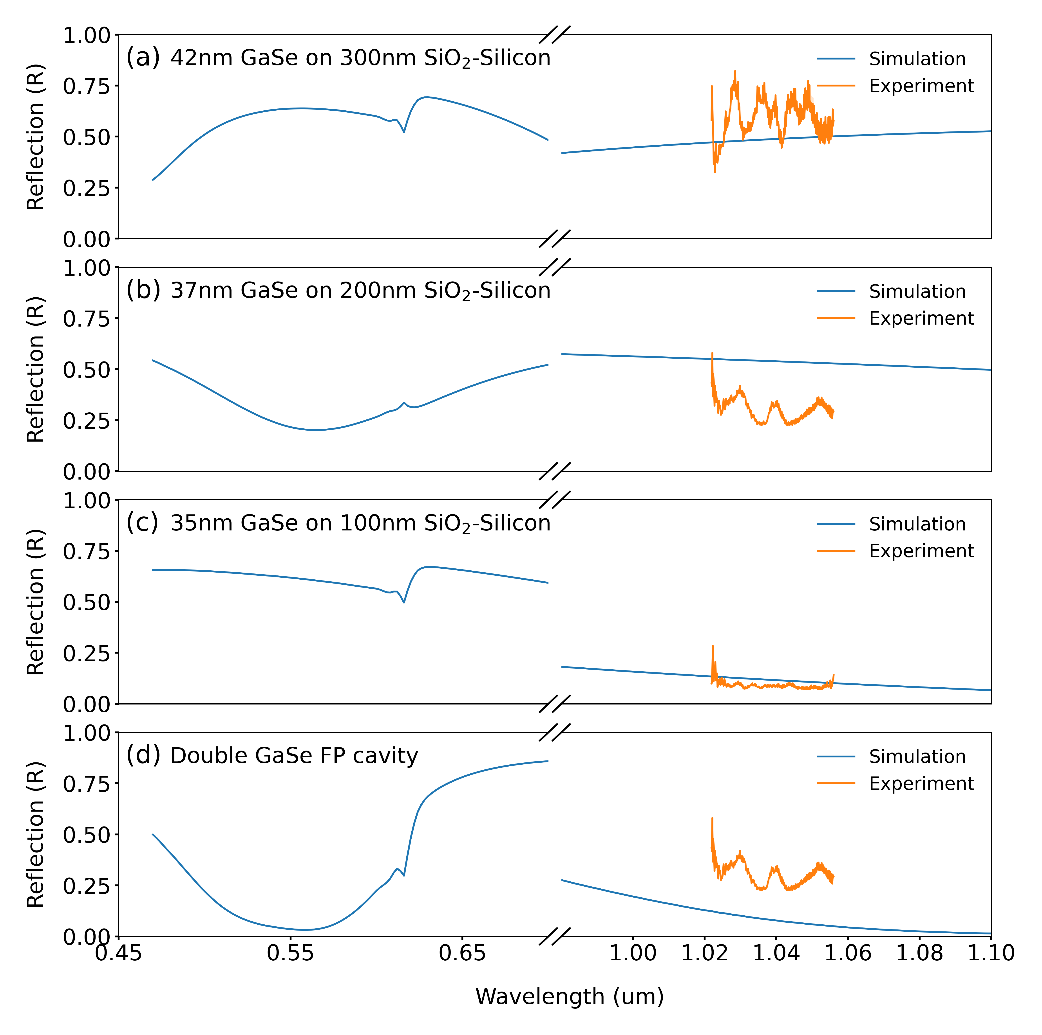


Figure S11 Reflectance spectrum of multilayer GaSe flakes of (a-c) single GaSe Fabry-Perot (d) and double GaSe Fabry-perot structure. Simulation result is expanded to incorporate the reflectance at the fundamental as well the second harmonic wavelength. Optical properties for the various materials in the stack are obtained from refs. [2-5].

**References:**

1. Kudryavtsev, A.V., Lavrov, S.D., Shestakova, A.P., Kulyuk, L.L. and Mishina, E.D., 2016. Second harmonic generation in nanoscale films of transition metal dichalcogenide: Accounting for multipath interference. AIP Advances, 6(9), p.095306.Boyd, R.W., 2020. Nonlinear optics. Academic press.
2. Palik, E.D. ed., 1998. Handbook of optical constants of solids (Vol. 3). Academic press.
3. Beadie, G., Brindza, M., Flynn, R.A., Rosenberg, A. and Shirk, J.S., 2015. Refractive index measurements of poly (methyl methacrylate)(PMMA) from 0.4–1.6 μm. Applied optics, 54(31), pp.F139-F143.
4. Le Toullec, R., Piccioli, N., Mejatty, M. and Balkanski, M., 1977. Optical constants of ε-GaSe. Il Nuovo Cimento B (1971-1996), 38(2), pp.159-167.
5. Kato, K., Tanno, F. and Umemura, N., 2013. Sellmeier and thermo-optic dispersion formulas for GaSe (Revisited). Applied optics, 52(11), pp.2325-2328.
